# Multifaceted Role of Sialylation in Prion Diseases

**DOI:** 10.3389/fnins.2016.00358

**Published:** 2016-08-08

**Authors:** Ilia V. Baskakov, Elizaveta Katorcha

**Affiliations:** Department of Anatomy and Neurobiology, Center for Biomedical Engineering and Technology, University of Maryland School of MedicineBaltimore, MD, USA

**Keywords:** prions, prion disease, amyloid, sialic acid, sialylation, species barrier, sialyltransferase, neuraminidase

## Abstract

Mammalian prion or PrP^Sc^ is a proteinaceous infectious agent that consists of a misfolded, self-replicating state of a sialoglycoprotein called the prion protein, or PrP^C^. Sialylation of the prion protein N-linked glycans was discovered more than 30 years ago, yet the role of sialylation in prion pathogenesis remains poorly understood. Recent years have witnessed extraordinary growth in interest in sialylation and established a critical role for sialic acids in host invasion and host-pathogen interactions. This review article summarizes current knowledge on the role of sialylation of the prion protein in prion diseases. First, we discuss the correlation between sialylation of PrP^Sc^ glycans and prion infectivity and describe the factors that control sialylation of PrP^Sc^. Second, we explain how glycan sialylation contributes to the prion replication barrier, defines strain-specific glycoform ratios, and imposes constraints for PrP^Sc^ structure. Third, several topics, including a possible role for sialylation in animal-to-human prion transmission, prion lymphotropism, toxicity, strain interference, and normal function of PrP^C^, are critically reviewed. Finally, a metabolic hypothesis on the role of sialylation in the etiology of sporadic prion diseases is proposed.

## Introduction

Prions or PrP^Sc^ are proteinaceous infectious agents that consist of misfolded, self-replicating states of a sialoglycoprotein called the prion protein or PrP^C^ (Prusiner, 1982; Legname et al., 2004). Prions cause prion diseases, a family of transmissible neurodegenerative maladies that have no treatment and are 100% lethal (Prusiner, 1998). Prions replicate by recruiting and converting PrP^C^ molecules expressed by a host into misfolded PrP^Sc^ states (Cohen and Prusiner, 1998). In the PrP^Sc^ state, the prion protein can acquire conformationally distinct self-replicating states referred to as prion strains, which elicit different, strain-specific disease phenotypes (Thomzig et al., 2004; Spassov et al., 2006; Morales et al., 2016). While the fact that PrP^Sc^ is sialylated has been known for more than 30 years (Bolton et al., 1985), little is known about the role sialylation plays in prion diseases. This review article summarizes current knowledge on the role of sialylation of the prion protein in prion diseases.

## Many amyloidogenic proteins exhibit prion-like behavior, but prions are unique

In recent years, convincing evidence was put in place illustrating that prion-like propagation of misfolded protein states is not limited to the prion protein (Jucker and Walker, 2013; Walker and Jucker, 2015). A number of amyloidogenic proteins or peptides, including Aβ, α-synuclein, tau, huntingtin, which are associated with a range of age-dependent neurodegenerative diseases, can also spread from cell to cell or be transmitted from animal to animal or human to animal in a prion-like fashion (Soto et al., 2006; Walker and Jucker, 2015). Even more striking, these amyloidogenic proteins can acquire several alternative disease-associated self-replicating states within the same amino acid sequence that recapitulates the prion strain phenomenon (Aguzzi, 2014; Stöhr et al., 2014; Watts et al., 2014).

While non-prion amyloidogenic proteins display certain characteristics of prion-like replication, several aspects make PrP^Sc^ unique. First, only PrP^Sc^ can be transmitted between organisms or species via natural routes (Brown and Gajdusek, 1991; Miller and Williams, 2004). Second, like microbial or viral agents, PrP^Sc^ shows incredibly high titers of up to 10^10.5^ infectious units per g of tissues in animal assays or 10^13^ units per g using *in vitro* assays (Makarava et al., 2012b). Such titers exceed by far those reported for other amyloidogenic proteins. Because different hosts are used for establishing titers (wild type vs. transgenic mice), direct comparison of prion titers with those displayed by non-prion amyloidogenic proteins should be done with caution. Keeping this in mind, 10^6^ was found to be the highest dilution of brain material with Aβ deposits formed in tg2576 mice that was able to seed Aβ misfolding in the same mouse line (Morales et al., 2015). Because Tg2576 mice is a transgenic line that overexpresses the Amyloid Precursor Protein harboring the Swedish mutation and shows spontaneous plaque formation with age, the titers established in Tg2576 might be overestimated. Third, PrP^Sc^-infected animals typically show a very robust course of disease progression characterized by a well-defined set of clinical symptoms, precise incubation time to disease, and a strict dependence of incubation time on dose. Fourth, in addition to the CNS, PrP^Sc^ accumulates in peripheral tissues, including the lymphoreticular system (Hilton et al., 1998; Sigurdson et al., 1999; Andréoletti et al., 2000; Aguzzi et al., 2013). In fact, not only does PrP^Sc^ colonize secondary lymphoid organs (SLOs), it replicates in SLOs autonomously from the CNS (Brown et al., 1999; Montrasio et al., 2000; Kujala et al., 2011; McCulloch et al., 2011). More surprisingly, despite low expression levels of PrP^C^ in SLOs, SLOs are more permissive to prions than the CNS (Béringue et al., 2012; Halliez et al., 2014). As such, SLOs represent silent reservoirs of infection, where prions could hide undetected in human populations while imposing a high risk of transmission through surgery, organ or blood donation (Hilton et al., 2004; Peden et al., 2004, 2010; Wroe et al., 2006; Bishop et al., 2013). The events triggered by peripheral prion infection sets prions aside from all other known types of pathogens as well. Whereas most bacteria, parasites, and viruses trigger innate and adaptive immune responses, the mammalian immune system appears to be remarkably tolerant to prions (Aguzzi et al., 2003).

## Introduction to sialylation

**Figure 1:**
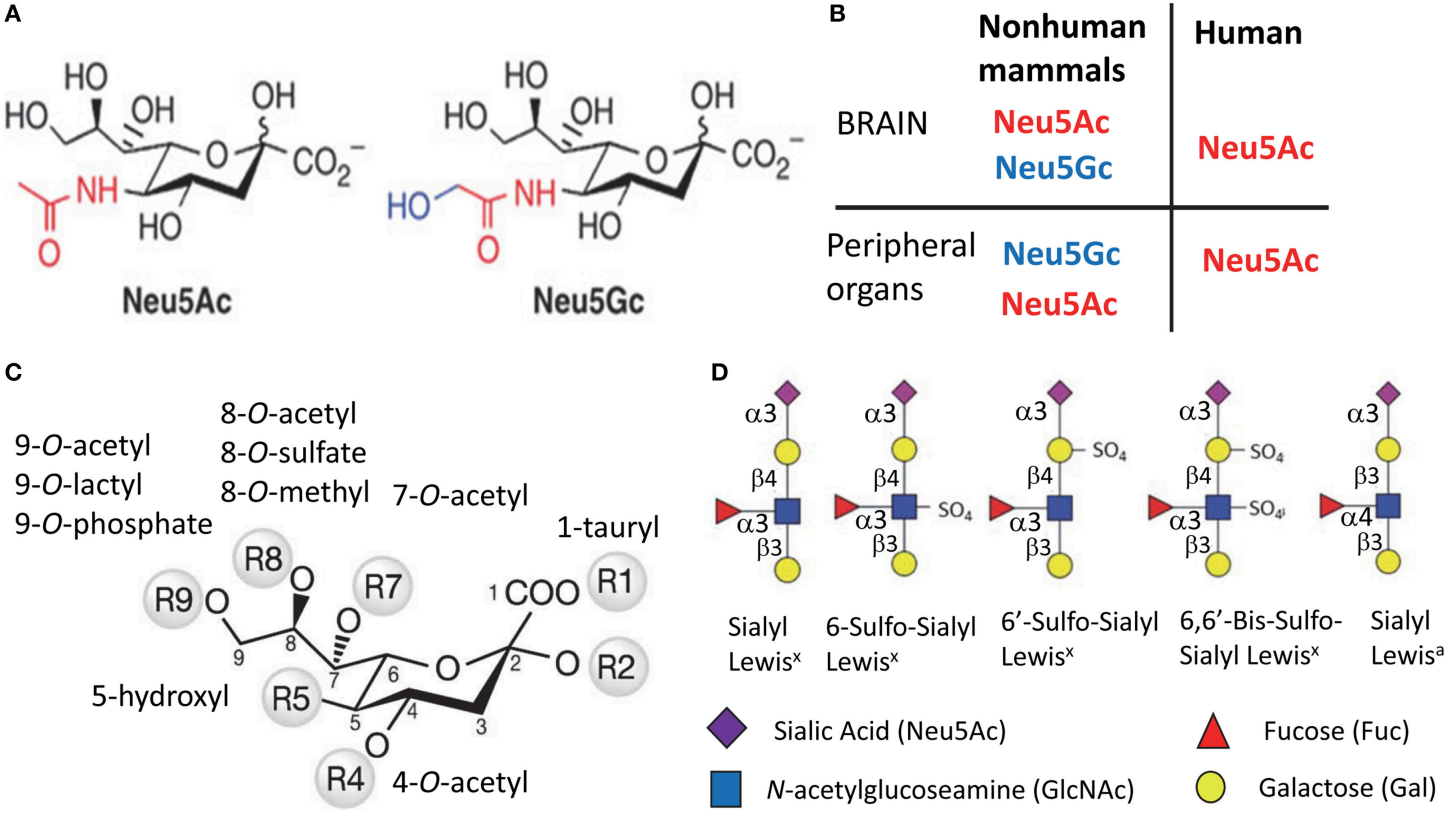
**Structural diversity of Sias**. Structures of two most common types of Sias, Neu5Ac, and Neu5Gc **(A)**, and a diagram illustrating the differences in Sias synthesized in humans vs. non-human mammals **(B)**. Structural diversity of Sias epitopes are achieved via naturally occurring modifications of Sias at 1-, 4-, 5-, 7-, 8-, or 9-carbon positions **(C)** and/or variations due to sulfation of galactose and *N*-acetylglucosamine that produce several Lewis glycoepitope families **(D)**. Panel **(D)** shows only a small subset of possible sulfated variants.

Sialic acids (Sias) are a family of 9-carbon containing acidic monosaccharides that are found in terminal positions of N- and O-linked glycans of glycoproteins or glycolipids (Figure 1A) (Varki, 1999). Glycan sialylation is controlled by two groups of enzymes: sialyltransferases (STs) and sialidases (NEUs) (Audry et al., 2011; Miyagi and Yamaguchi, 2012). STs transfer sialic acids to the terminal positions of glycans. This process takes place in the trans-Golgi and involves 20 mammalian STs (Audry et al., 2011). STs are divided into four families according to the type of linkages synthesized (α2-3, α2-6, α2-8, or α2-9) and the selectivity toward N- or O-linked glycans (Takashima, 2008; Audry et al., 2011). NEUs, on the other hand, remove Sias from glycans. Four NEUs are found in mammals, they are expressed in a tissue-specific manner and display differences in cellular localization (Monti et al., 2010; Miyagi and Yamaguchi, 2012; Pshezhetsky and Ashmarina, 2013).

Humans can synthesize only one type of Sias, which is N-acetylneuraminic acid (Neu5Ac) (Varki, 2010) (Figures 1A,B). With the exception of the ferret (Ng et al., 2014), the rest of mammalian species produce two types of Sias. Neu5Ac is the predominant type that is synthesized in a brain, whereas Neu5Ac and N-glycolylneuraminic acid (Neu5Gc) are synthesized by peripheral organs (Varki, 1999) (Figure 1B). The deficiency in synthesis of Neu5Gc in humans is due to an irreversible mutation in the gene encoding cytidine monophosphate N-acetylneuraminic acid hydroxylase (an enzyme that synthesize Neu5Gc from Neu5Ac) that occurred during evolution from primates to humans (Varki, 2010). Like humans, ferrets can produce only Neu5Ac (Ng et al., 2014). While humans lack the ability to synthesize Neu5Gc, it can be incorporated metabolically into human cells from diet (Samraj et al., 2015).

Sias on cell surface glycans and glycolipids form diverse structural epitopes that are involved in a number of cellular functions. The structural diversity of Sia epitopes is produced via several mechanisms. First, Sias can be attached to galactose or N-acetylgalactosamine of glycans via α2-3, α2-6, α2-8, or α2-9 linkages (Varki, 1999). Second, various natural substitutes including *O*-acetyl, *N*-glycolyl, *O*-lactyl, *O*-sulfate, *O*-phosphate, tauryl, hydroxyl, or *O*-methyl can be synthesized on carbons of Sias at 1-, 4-, 5-, 7-, 8-, or 9-positions, where O-acetyl being the most common substitute (Figure 1C). (Schauer et al., 2011). Third, in combination with Sias other groups including sulfate and fucose are involved in forming functional glycan epitopes including Sialyl Lewis^x^, Sialyl Lewis^a^, 6′Sulfo-Sialyl Lewis^x^ (Figure 1D) (Fukuda et al., 1999). Fourth, complex glycans can exhibit several branching patterns that contribute to variations in density of Sia residues, a factor important for binding of multivalent ligands.

Sias are abundant on the surfaces of all mammalian cell types with an estimated local concentration on the cell surface glycocalyx approaching 100 mM (Collins et al., 2004). Recent years witnessed an extraordinary rise in interest to sialylation and established its role in host-pathogen interactions and communication between cells of immune system (Varki, 2008, 2010). Sias on the surface of mammalian cells act as a part of “self-associated molecular pattern” helping the immune system to recognize “self” from “altered self” or “non-self” (Varki, 2008; Brown and Neher, 2014). A decline in Sia content represents one of the molecular signatures of “apoptotic-cell-associated molecular patterns” found in apoptotic or aging cells (Savill et al., 2002; Brown and Neher, 2014). Removal of Sias from cell surface glycans exposes galactose residues that generate “eat me” signals for professional and non-professional macrophages. Examples include clearance of erythrocytes or platelets with reduced sialic acid residues by Kupffer cells (Aminoff et al., 1977; Jansen et al., 2012) or neurons by microglia (Linnartz et al., 2012; Linnartz-Gerlach et al., 2016). Lack of Sias on the cell surface is also a part of the “pathogen-associated molecular pattern” or PAMPs used by mammalian immune systems to recognize pathogens or asialoglycoproteins that need to be removed (Varki, 2008).

## N-linked glycans on PrP^C^ and PrP^Sc^ are sialylated

PrP^C^ is posttranslationally modified with up to two N-linked glycans and a GPI anchor (Stahl et al., 1987; Endo et al., 1989). In PrP^C^, Sias are linked to the terminal positions of the two N-linked glycans via α2-3 or α2-6 linkages with the majority being linked via α2-6 (Turk et al., 1988; Endo et al., 1989; Stimson et al., 1999). Each of the two glycans has up to five terminal Sias (Endo et al., 1989; Rudd et al., 1999). Variation in structure and composition of N-linked glycans give rise to more than 400 different PrP^C^ glycoforms (Endo et al., 1989; Stimson et al., 1999). Upon conversion of PrP^C^ into PrP^Sc^, the sialylated glycans and GPI are carried over, giving rise to sialylated PrP^Sc^ (Bolton et al., 1985; Stahl et al., 1993; Rudd et al., 1999).

In the absence of posttranslational modifications, the theoretical pI of the full-length mouse prion protein is expected to be 9.6 and the estimated charge at pH 7.5 is +9.5 (Katorcha et al., 2014). However, due to glycan sialylation, the actual pI of PrP molecules was found to be highly heterogeneous and spread from pH 9.6 to acidic pH (DeArmond et al., 1999; Katorcha et al., 2014). In intact PrP^Sc^ particles, the glycans are believed to be directed outwards, with the terminal sialic acid residues located at the interface with the extracellular environment or solvent (Wille et al., 2002; Govaerts et al., 2004; Requena and Wille, 2014).

## Sialylation of GPI anchor

In addition to sialylation of N-linked glycans, a single Sia could be also present on the GPI anchor of PrP^C^ (Stahl et al., 1992). The question regarding sialylation status of GPIs within PrP^Sc^ has been controversial. As judged from mass-spectroscopy analysis of hamster-adapted prion strains Sc237 and 139H, approximately 30% of GPIs of brain-derived PrP^Sc^ were found to be sialylated (Stahl et al., 1992). Moreover, the composition of GPIs within PrP^Sc^ was found to be similar to that of PrP^C^ (Stahl et al., 1992). In contrast, recent studies by Bate and coauthors claimed that PrP^C^ with asialo-GPIs were not convertible into PrP^Sc^ and, even more, inhibited conversion of PrP^C^ with sialo-GPIs into PrP^Sc^ (Bate et al., 2016b). To arrive at this conclusion, a cell painting technique was used for administering PrP^C^ with sialo- or asialo-GPIs to cultured N2a neuroblastoma cells or primary neurons. Our recent studies that examined tissues from mice or hamsters infected with five prion strains or prion infected N2a cells or C2C12 myotube cells revealed that PrP^C^ molecules with both sialo- and asialo-GPIs were recruited into PrP^Sc^ (Katorcha et al., 2016). Notably, the proportion of sialo- vs. asialo-GPIs within PrP^Sc^ was found to be controlled by host, tissue, and cell type, but not prion strain (Katorcha et al., 2016).

In a series of other studies that also employed cell painting techniques, Bate and coauthors suggested that toxicity triggered by PrP^Sc^ is dependent on the sialylation status of GPI anchor within PrP^C^, as clustering of PrP^C^ molecules with sialo-GPIs led to activation of cytoplasmic phospholipase A2 and synapse damage (Bate and Williams, 2012b). Moreover, sialylation status of GPIs was found to modify the local environment of PrP^C^ where the greater amounts of sialylated gangliosides and cholesterol were found in rafts surrounding PrP^C^ with asialo-GPIs relative to PrP^C^ with sialo-GPIs (Bate et al., 2016b). In addition, sialo-GPIs were found to target exogenous PrP^C^ to synapses of neurons derived from the prion protein knockout mice (Bate et al., 2016a). Because cell painting technique was used in aforementioned studies, the questions whether the conclusions reached by using PrP^C^ exogenously added to cells are valid for PrP^C^ expressed by a cell or in animals have to be addressed.

## The effect of sialylation of PrP^Sc^ glycans on prion infectivity and disease outcome

**Figure 2:**
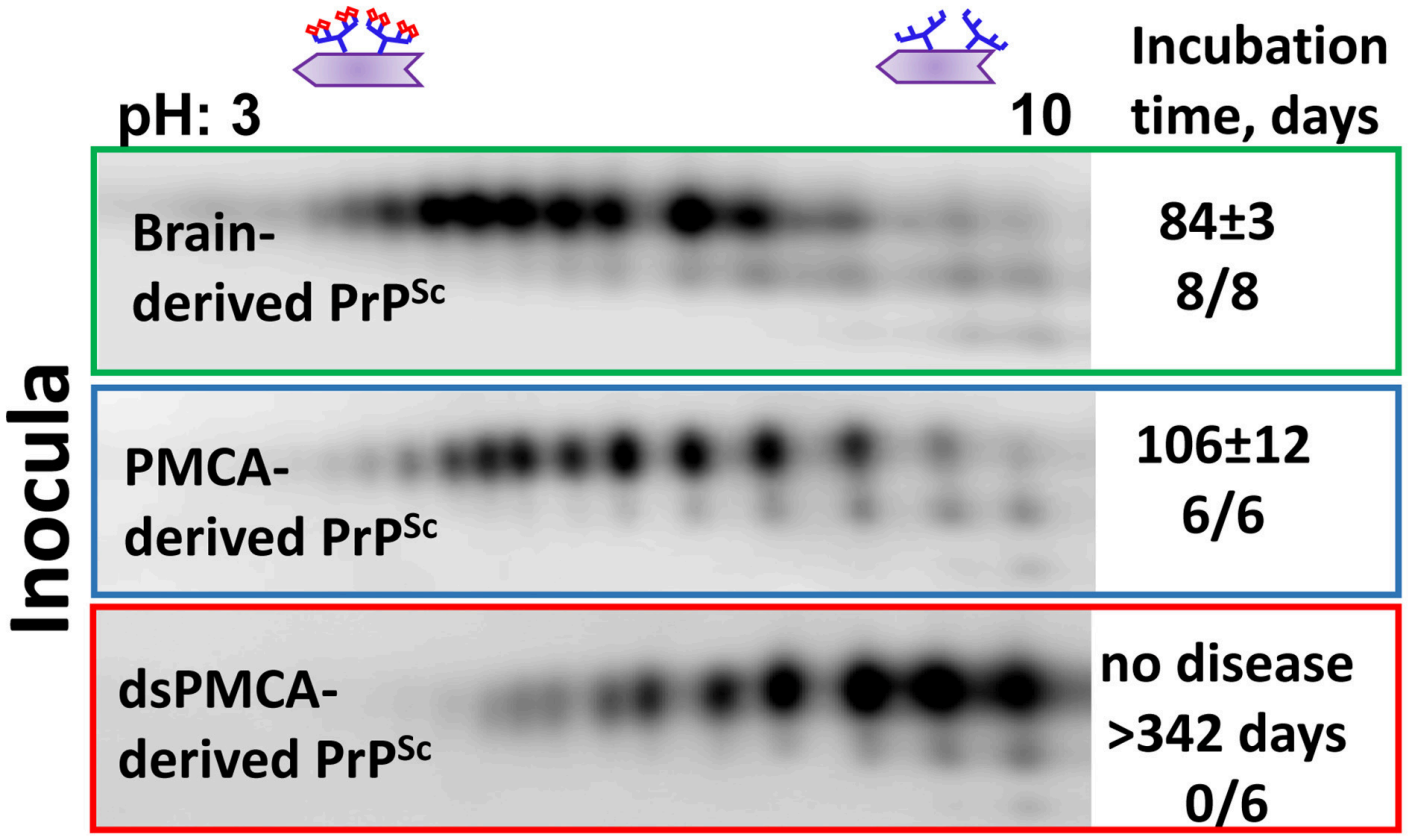
**Analysis of sialylation status of brain-, PMCAb-, and dsPMCAb-derived PrP^**Sc**^ for 263K strain using 2D western blots**. Incubation time to disease and number of animals that developed clinical disease out of total number of animals is shown on the right. The data represented here is a modification of the figure from previously published manuscript (Katorcha et al., 2014).

Recent studies from our laboratory revealed that PrP^Sc^ with reduced sialylation levels does not induce prion disease in wild type animals (Katorcha et al., 2014). To produce PrP^Sc^ with reduced sialylation, Protein Misfolding Cyclic Amplification with beads (PMCAb) was conducted using PrP^C^ as a substrate that was partially desialylated by treatment with sialidases (dsPMCAb). As a reference, PrP^Sc^ was also produced in PMCAb reactions conducted with non-treated PrP^C^. Both types of reactions were seeded with hamster scrapie strain 263K. All animals inoculated with brain-derived 263K developed clinical signs and showed substantial amounts of PrP^Sc^ in their brains (Figure 2). Animals inoculated with PMCAb-derived 263K developed disease at slightly longer incubation times relative to the control group that received brain-derived PrP^Sc^. Such delay is attributed to a moderate shift in the sialylation pattern of PMCAb-derived 263K relative to that of brain-derived 263K (Figure 2). In the course of PMCAb, PrP^C^ molecules with low sialylation status were preferentially recruited into PrP^Sc^, producing a shift in sialylation status of PMCAb-derived material (Katorcha et al., 2014). Remarkably, no animals inoculated with dsPMCAb-derived material developed the disease (Katorcha et al., 2014) (Figure 2). Moreover, no PrP^Sc^ was detected in brains or spleens of animals from these groups by Western blot or serial PMCAb, arguing that the animals injected with dsPMCAb material were not subclinical carriers of scrapie. Because exposed galactose residues are believed to generate “eat me” signals, we propose that dsPMCAb-derived material is degraded rapidly due to an increase in amounts of terminal galactose as a result of partial removal of sialic acid residues. This hypothesis has to be tested in future studies.

## Factors that control sialylation of PrP^Sc^

The sialylation status of PrP^Sc^ appears to be of paramount importance to prion infectivity; therefore, dissecting the mechanisms that control sialylation status of PrP^Sc^ is of great interest. Because PrP^Sc^ arises from PrP^C^ via changes in its conformation, it is important to understand the mechanisms that control sialylation of PrP^C^.

### Sialyltransferases and sialidases

**Figure 3:**
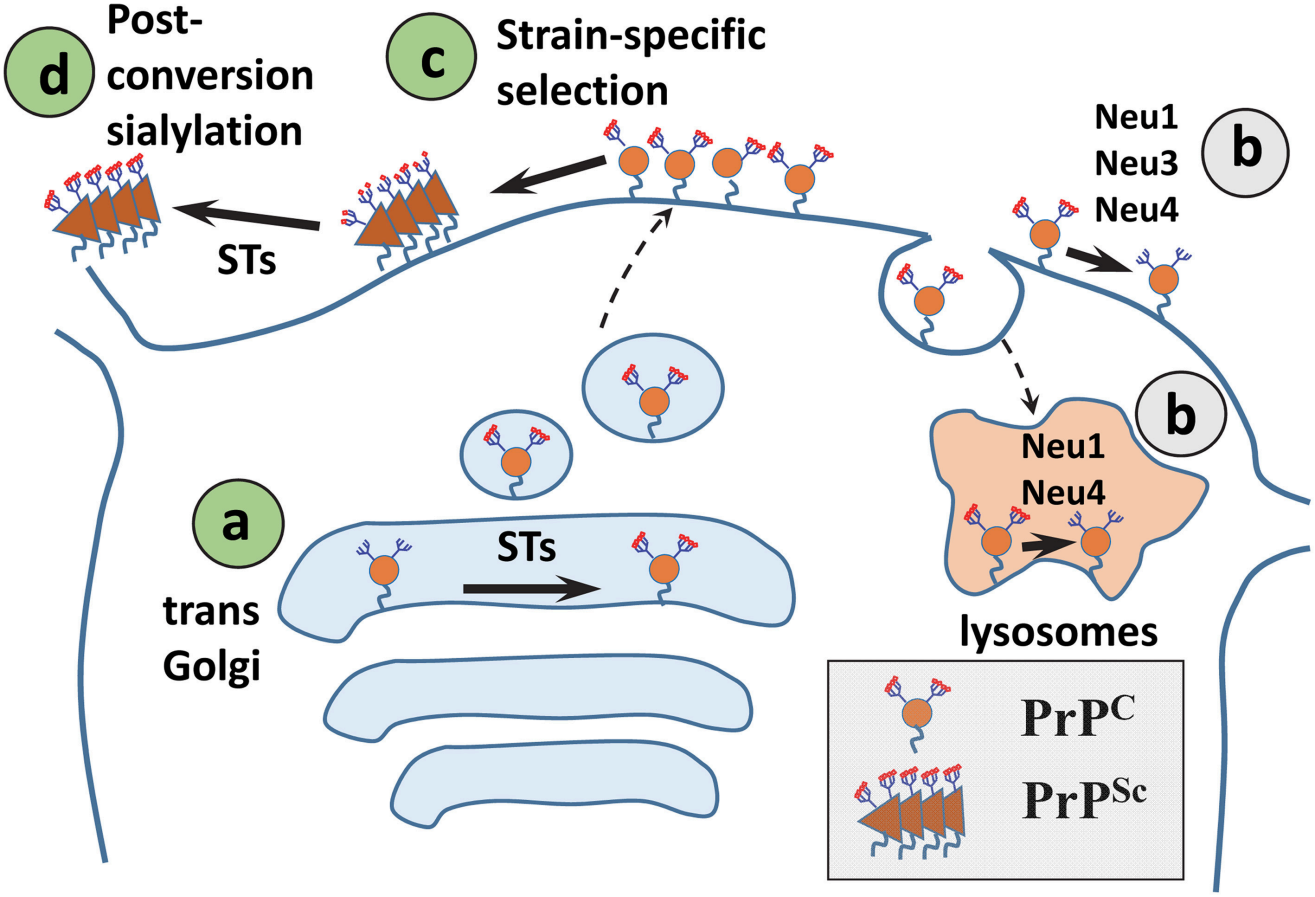
**A diagram illustrating mechanisms that control sialylation of PrP^**Sc**^. (A)** The sialylation status of PrP^C^ is controlled by STs in the trans-Golgi. **(B)** NEUs do not appear to affect the steady-state sialylation level of PrP^C^, presumably because desialylated PrP^C^ is degraded rapidly. **(C)** Sialoglycoforms of PrP^C^ are recruitment into PrP^Sc^ selectively according to their sialylation status and in a strain-specific manner. **(D)** In SLOs, PrP^Sc^ is a subject of post-conversion sialylation by STs. Sias are shown as red diamonds.

The steady-state level of sialylation in a cell is controlled by two groups of enzymes: STs and NEUs. Of the four NEUs expressed in mammals, NEU1 localizes to the lysosomes and cell surface, NEU2 is found in the cytoplasm and is expressed in muscles, NEU3 is at the plasma membrane, and NEU4 is associated with mitochondria, lysosomes, and ER, but can also be recruited to the cell surface (Monti et al., 2010; Miyagi and Yamaguchi, 2012; Pshezhetsky and Ashmarina, 2013). Because PrP^C^ is localized at the cell surface and in endocytic/lysosomal compartments, three out of four NEUs (NEU1, NEU3, or NEU4) could be involved in regulating sialylation of PrP^C^. Surprisingly, brain materials from *Neu1, Neu3, Neu4* knockout, or *Neu3/Neu4* double knockout mice showed no differences in sialylation status of PrP^C^ or its proteolytic fragment C1 in comparison to the corresponding wild type controls (Katorcha et al., 2015a). Moreover, suppressing NEU activity using the general inhibitor DANA did not change the sialylation of PrP^C^/C1 in neurobalstoma N2a cells, but did alter the global sialylation status (Katorcha et al., 2015a). These results suggested that upon removal of Sias from PrP^C^ by cellular NEUs, PrP^C^ molecules are degraded very fast and do not contribute to the steady-state pool of PrP^C^ (Figure 3). If desialylation results in fast degradation, sialidase deficiency is expected to cause accumulation of PrP^C^ and/or C1. Indeed, higher amounts of total PrP signal (PrP^C^ plus C1) was observed in brains of *Neu1, Neu3*, and *Neu4* knockout mice as expected (Katorcha et al., 2015a). An alternative hypothesis proposes that PrP^C^/C1 are not targeted by NEUs as a substrate.

Modulating the activity of STs instead of NEUs may offer a more effective strategy for controlling the sialylation status of PrP^C^. A general inhibitor of STs 3F_ax_-Neu5Ac was found to reduce the sialylation level of PrP^C^ in N2a cells (Katorcha et al., 2015a). Out of 20 mammalian STs, five STs exhibit substrate specificity for sialylation of N-linked glycans via α2-3 or α2-6 linkages, the type of linkages found in PrP^C^ and PrP^Sc^. Three out of the aforementioned five STs that supposedly have PrP-directed sialylation activity belong to the ST3 family (ST3Gal3, ST3Gal4, and ST3Gal6) and sialylate N-linked glycans via α2-3 linkages. The remaining two STs belong to the ST6 family (ST6Gal1 and ST6Gal2) and sialylate N-linked glycans via α2-6 linkages (Takashima, 2008; Audry et al., 2011). ST6Gal2 is found predominantly in fetal brain, whereas ST6Gal1 is expressed throughout the organism including the CNS (Takashima et al., 2002, 2003). Knocking out ST6Gal1 was found to reduce dramatically the amounts of α2-6 linked sialic acids in peripheral organs and the CNS (Martin et al., 2002), suggesting that ST6Gal1 is the main enzyme responsible for α2-6-linked sialylation and that its function is not redundant. In mice infected with prions expression of 165 glycosylation-related genes was analyzed (Guillerme-Bosselut et al., 2009). Among them, the expression levels of ST6Gal1 mRNA was found to be upregulated by ~3-fold in brain and spleen at the terminal stages of the disease that might reflect the pro-inflammatory response to the disease (Guillerme-Bosselut et al., 2009). It is not known whether the sialylation status of PrP^C^ and/or PrP^Sc^ changes due to upregulation of ST6Gal1 in the course of prion infection.

### Strain-specific selection of PrP^C^ sialoglycoforms

**Figure 4:**
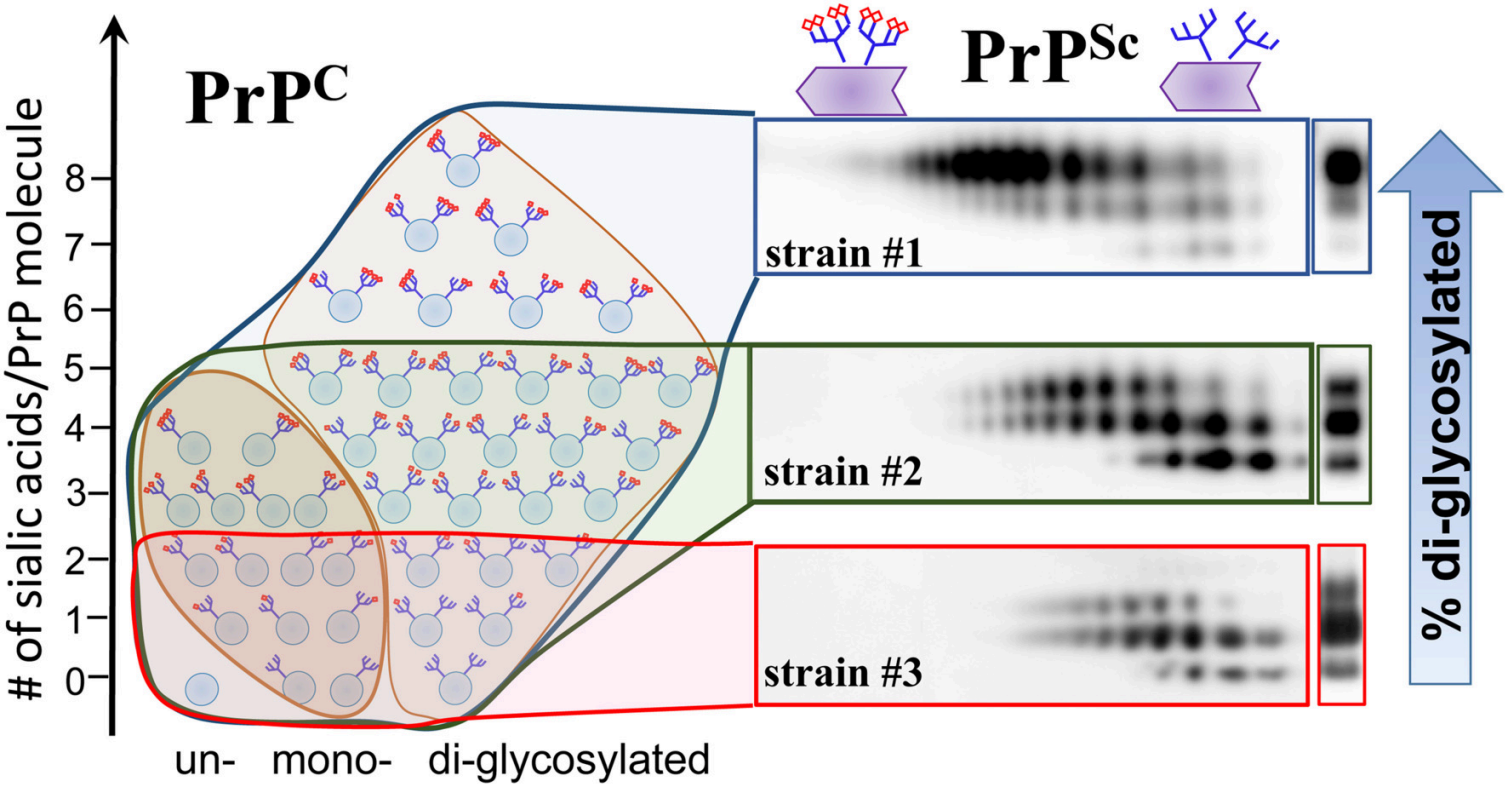
**Schematic diagram illustrating that PrP^**Sc**^ strains recruit PrP^**C**^ isoforms selectively according to PrP^**C**^ sialylation status**. While strain #1 recruits sialoglycoforms of PrP^C^ without noticeable preferences, hypersialylated PrP^C^ are preferentially excluded from the strain the #2 and even more so from strain #3. As a result of strain-specific exclusion of highly sialylated PrP^C^ (illustrated by the 2D Western blots), the ratios of di- vs. mono-glycoforms within PrP^Sc^ changes in a strain-specific manner, as shown by 1D Western blots (right hand side). PrP^C^ molecules are shown as blue circles and sialic acid residues—as red diamonds.

PrP^C^ molecules are heterogeneous with respect to the sialylation levels of their N-linked glycans ranging from hyposialylated to hypersialylated (DeArmond et al., 1999; Katorcha et al., 2014; Schmitz et al., 2014). Using hamster strain 263K, Rudd and coauthors showed that in brain, the relative populations of sialyloglycoforms of PrP^Sc^ were very similar to those of PrP^C^ (Rudd et al., 1999). This result led to the conclusion that PrP^C^ sialoforms are recruited into PrP^Sc^ proportionally to their relative presentation in a cell (Rudd et al., 1999). Recent studies examined a panel of mouse and hamster strains and discovered a remarkable pattern: hypersialylated PrP^C^ molecules were partially excluded from PrP^Sc^ (Katorcha et al., 2015b) (Figure 3). The degree to which hypersialylated PrP^C^ were excluded was strain-specific and found to be minimal for 263K, explaining the findings by Rudd et al. Strain-specific exclusion suggests that some strains can accommodate heavily sialylated PrP^C^ molecules better than others (Figure 4).

### Tissue-specific post-conversion sialylation of PrP^Sc^

Upon prion transmission via peripheral routes, PrP^Sc^ is first sequestered by SLOs, including spleen and lymph nodes, prior to invasion of the CNS (Huang et al., 2002; Takakura et al., 2011; Castro-Seoane et al., 2012; Michel et al., 2012). Moreover, PrP^Sc^ replicates in SLOs independently of replication in the CNS (Brown et al., 1999; Montrasio et al., 2000; Kujala et al., 2011; McCulloch et al., 2011). Recent studies revealed that spleen-derived PrP^Sc^ is considerably more sialylated than brain-derived PrP^Sc^ (Srivastava et al., 2015). Enhanced sialylation of PrP^Sc^ in SLOs was observed regardless of prion strain, host species, or inoculation route (Srivastava et al., 2015). Remarkably, enhanced sialylation of PrP^Sc^ was not due to enhanced sialylation of PrP^C^ expressed in SLOs, but appears to be due to post-conversion sialylation of PrP^Sc^ in SLOs by extracellular STs (Figure 3). While STs are traditionally believed to localize within the trans-Golgi (Harduin-Lepers et al., 2001), a number of studies reported ST activity in circulation or on surfaces of the cells of the immune system including polymorphonuclear leukocytes, monocyte-derived dendritic cells, lymphocytes, and T cells (Gross et al., 1996; Kaufmann et al., 1999; Schwartz-Albiez et al., 2004; Rifat et al., 2008; Cabral et al., 2010; Nasirikenari et al., 2014). Consistent with the hypothesis that extracellular STs are involved in enhancing sialylation of PrP^Sc^, the sialylation status of foreign PrP^Sc^ acquired via peripheral exposure changed with colonization of SLOs (Srivastava et al., 2015). Moreover, enhanced sialylation of PrP^Sc^ was recapitulated *in vitro* by incubating brain-derived PrP^Sc^ with primary splenocytes or cultured macrophage RAW 264.7 cells (Srivastava et al., 2015). General inhibitors of STs suppressed enhanced sialylation of PrP^Sc^ (Srivastava et al., 2015). Thus, post-conversion sialylation is likely to camouflage PrP^Sc^ in SLOs. It would be interesting to test whether enhanced sialylation of PrP^Sc^ accounts for the high permissiveness of SLOs to prion infection.

## Sialylation contributes to prion replication barrier

The conformational transition from PrP^C^ into PrP^Sc^ is regulated by a large energy barrier that controls the prion conversion rate (Baskakov et al., 2001). Due to the large energy barrier, the spontaneous conversion of PrP^C^ into PrP^Sc^ is very rare, explaining the low occurrence rates of sporadic prion diseases (Cohen and Prusiner, 1998). The magnitude of the energy barrier is attributed to the energy needed to unfold PrP^C^ (Baskakov et al., 2001). Recent studies that employed PMCAb proposed that electrostatic repulsions between sialic acid residues also create structural constraints for PrP^Sc^ replication and contribute to the replication barrier (Katorcha et al., 2015b). In PrP^Sc^ particles, glycans are directed outward where the terminal sialic acid residues create a dense negative charge on the PrP^Sc^ surface (Wille et al., 2002; Govaerts et al., 2004; Requena and Wille, 2014). Because of strain-specific differences in PrP^Sc^ structures, the contribution of sialic residues to the barrier is expected to be strain-specific. Indeed, several lines of evidence support this hypothesis. First, heavily sialylated PrP^C^ molecules were found to be partially excluded from conversion into PrP^Sc^, and the degree of exclusion was found to be strain-specific (Katorcha et al., 2015b). Second, desialylation of PrP^C^ by enzymatic treatment with sialidases removed the structural constraints and increased the rates of replication of PrP^Sc^ in PMCAb. The increase in replication rates was strain-specific, too, ranging from 20- to 10^6^-fold (Katorcha et al., 2014, 2015b). Third, desialylation of PrP^C^ was also found to considerably reduce the barrier in cross-seeded replication of PrP^Sc^ in PMCAb reactions (Katorcha et al., 2014). Together, these data suggest that the replication barrier attributable to glycan sialylation is universal, i.e., not only does it control the rate of prion replication within the same host but also the barrier associated with prion transmission between different species. The electrostatic repulsion between sialylated glycans of nascent PrP^Sc^ and the size of glycans are expected to impose a negative impact on the thermodynamic stability of PrP^Sc^ particles. This negative impact has to be counteracted by other forces that stabilize the packing of polypeptide chains within PrP^Sc^ particles. Notably, while thermodynamic stability of PrP^Sc^ varies depending on the strain-specific structure (Peretz et al., 2001; Colby et al., 2009; Ayers et al., 2011; Gonzalez-Montalban et al., 2011), the range of strain-specific thermodynamic stabilities of PrP^Sc^ is typically lower than those of amyloid fibrils generated *in vitro* from recombinant PrP (Sun et al., 2007, 2008). In part, such differences are likely due to electrostatic repulsion between sialylated glycans that recombinant PrP lacks.

Additional parameters have to be considered in discussing the effect of sialylation on the replication barrier *in vivo*. Because PrP^C^ glycans could be bi-, tri- or tetra-antennary, PrP^C^ molecules with the same number of sialic acid residues per molecule might have different number of terminal galactose that serves as “eat me” signal. PrP^C^ molecules with substantial levels of terminal galactose are expected to be degraded quickly. While heavily sialylated PrP^C^ molecules are excluded from conversion for conformational reasons, weakly sialylated PrP^C^ with bulky glycans might not be involved in replication either due to their fast degradation. Therefore, *in vivo* the size of glycans, sialylation levels, and the number of exposed galactose are likely to define the availability and eligibility of PrP^C^ as a substrate.

## Sialylation and strain-specific glycoform ratio

The glycoform ratio within PrP^Sc^ is considered to be one of the primary intrinsic characteristics of prion strains or PrP^Sc^ subtypes (reviewed in Lawson et al., 2005). While the mechanisms behind strain-specific selectivity in recruitment of glycoforms remain unknown, the glycoform ratios have been used in the prion field for strain typing and classification of CJD type (Collinge et al., 1996; Somerville, 1999). Recent studies that analyzed strain-specific sialylation patterns of PrP^Sc^ revealed that the strain-specific glycoform ratio is due to exclusion of heavily sialylated PrP^C^ molecules (Katorcha et al., 2015b) (Figure 4). Because diglycosylated PrP^C^ carry more sialic acid residues per molecule on average than mono- or unglycosylated PrP^C^, the preferential exclusion of heavily sialylated PrP^C^ is achieved via (i) selective recruitment of mono- and unglycosylated PrP^C^ at the expense of diglycosylated PrP^C^, and (ii) preferential exclusion of hypersialylated diglycosylated PrP^C^ (Katorcha et al., 2015b). In fact, a correlation between PrP^Sc^ sialylation status and the glycoform ratio exists (Katorcha et al., 2015b). Remarkably, when exposed to desialylated PrP^C^ as a substrate, prion strains lose strain-specific selectivity toward PrP^C^ glycoforms, and the glycoform ratio within PrP^Sc^ mirrors that of PrP^C^ (Katorcha et al., 2015b).

## N-linked glycans and PrP^Sc^ structure

**Figure 5:**
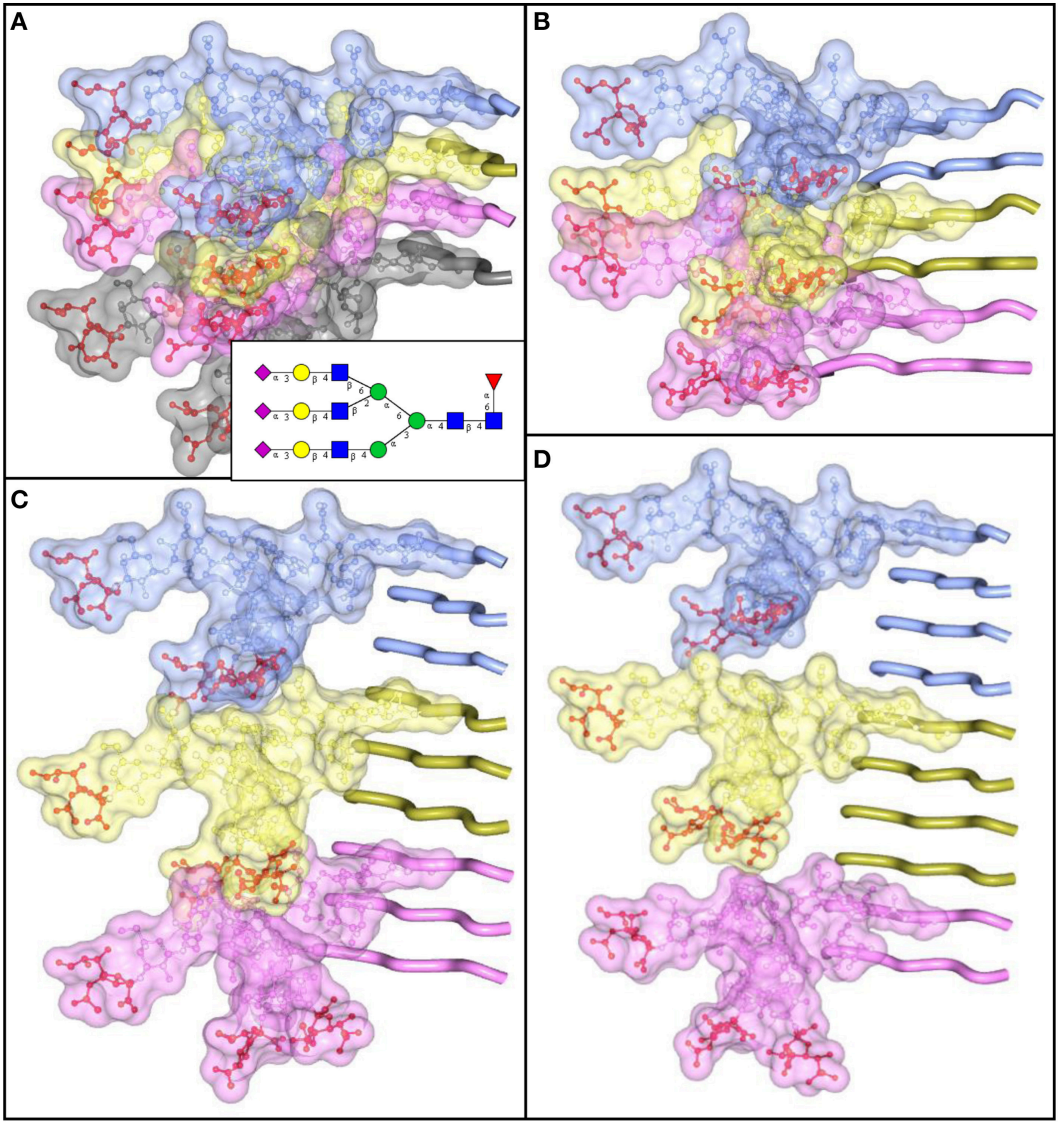
**N-linked glycans impose spatial constraints on folding patterns of PrP^**Sc**^**. Cross beta-sheet structures carrying tri-antennary N-glycans (shown in inset) on each neighboring beta-strand **(A)**, or every second **(B)**, third **(C)**, or fourth **(D)** beta-strand. Polypeptide chains are represented in the tube form, whereas N-glycans are represented in the ball-and-stick form. Each PrP molecule with corresponding N-glycan is of a different color. Sialic acid residues are colored in red; N-glycan electrostatic surfaces are semi-transparent. To model the dimension of cross-beta structures, the parallel beta-sheet model was adapted from PDB database entry 2RNM, an NMR structure for HET-s(218–289) prion in its amyloid form (Wasmer et al., 2008). Stretches of seven amino acid residues are shows for each beta strand without any change to the atomic coordinates. The structure of a tri-antennary N-linked glycan was taken from PDB entry 3QUM, a crystal structure of human prostate specific antigen (PSA) (Stura et al., 2011). Both calculations of electrostatic surfaces and generation of images were performed with CCP4MG software.

The density of sialylation and size of N-linked glycans impose considerable structural constraints, limiting the range of plausible structures for PrP^Sc^. Similar to amyloids formed by other amyloidogenic proteins or peptides, PrP^Sc^ exhibits a cross-β folding pattern (Wille et al., 2009; Ostapchenko et al., 2010), a key structural feature of amyloid states. However, the precise folding pattern of PrP molecules within PrP^Sc^ has been debated (reviewed in Requena and Wille, 2014). The recent PIRIBS model proposed that PrP^Sc^ consists of an in-register parallel β-sheet structure, in which each PrP molecule occupies a single layer within cross-β fibers (Groveman et al., 2014). This model is similar to those proposed earlier for amyloid fibrils formed by non-glycosylated recombinant PrP (Cobb et al., 2007; Tycko et al., 2010). Alternative models postulate that within PrP^Sc^ fibers each PrP molecule forms a multi-rung β-solenoid (Govaerts et al., 2004; Amenitsch et al., 2013). To discriminate between alternative models, we decided to determine the extent to which N-linked glycans can be accommodated within PrP^Sc^ folding patterns proposed by different models. According to the PIRIBS model, the glycans linked to the same amino acid residue on neighboring PrP molecules are separated by a distance of 4.7 Å (Figure 5A). For solenoid models, depending on the number of rungs formed by PrP molecules within the solenoid, the distance between glycans on neighboring PrP molecules could be 2 × 4.7 Å, 3 × 4.7 Å, or 4 × 4.7 Å for the solenoids consisted of 2, 3, or 4 rungs, respectively (Figures 5B–D). To model N-linked glycans of average size, we choose a tri-antennary glycan structure, since PrP^C^ and PrP^Sc^ are known to carry bi-, tri-, and tetra-antennary glycans (Endo et al., 1989; Rudd et al., 1999; Stimson et al., 1999). Substantial spatial overlap was found between glycans of neighboring PrP molecules, if the glycan linkages were separated by distances 4.7 Å or 2 × 4.7 Å (Figures 5A,B). Such spatial constraints argue strongly against PIRIBS or 2-rung solenoids as plausible models of PrP^Sc^. Minor spatial overlap was observed between neighboring glycans attached at the distance of 3 × 4.7 Å, and no overlap when the tri-antennary glycans were separated by 4 × 4.7 Å (Figures 5C,D). The minor overlap observed for 3-rung solenoid structures could be avoided if N-linked glycans are of smaller-sizes (bi-antennary) and/or oriented at various angles. The 2-rung solenoid structure would be still possible if glycosylated molecules alternated with non-glycosylated ones along PrP^Sc^ fibers. However, the percentage of non-glysoylated PrP molecules within PrP^Sc^ is known to be very small (Nishina et al., 2006; Katorcha et al., 2015b).

## Metabolic origin of sporadic prion diseases

According to the Braak staging hypothesis, in Alzheimer's and Parkinson diseases amyloid deposits and pathology originate in certain areas of the CNS and spread in a prion-like manner through the brain in disease-specific patterns (Braak and Braak, 1991). It is not known from which brain area sporadic CJD originates and whether it spreads in a specific pattern. Bearing in mind that sialylation controls the height of the conformational transition barrier (Katorcha et al., 2015b), it is reasonable to propose that the first spontaneous PrP^C^-to-PrP^Sc^ conversion events have a higher chance of occurring in brain areas that express PrP^C^ with glycans of small sizes and reduced sialylation levels and/or in individuals with deficient sialylation substrate. Consistent with the metabolic hypothesis, there is a decline in total sialic acid content as well as cell surface sialylation with age (Svennerholm et al., 1997). Notably, a polymorphism in β-secretase (BACE1), an enzyme that cleaves ST6Gal1, was recently shown to be a risk factor for sCJD suggesting that a link between sialylation and sCJD might exist (Calero et al., 2012). It would be interesting to test whether variations in BACE1 activity due to polymorphism contribute to stability and/or activity of ST6Gal1 in Golgi and affect sialylation status of PrP^C^.

## Animal-to-human prion transmission

Humans can only synthesize Neu5Ac, whereas Neu5Gc is the predominant type of Sias expressed in the periphery of mammals (Varki, 2010) (Figure 1B). The difference in the type of Sias expressed in the periphery of human and non-human mammals raises several important topics for discussion. First, it would be interesting to find out whether this difference contributes to the animal-to-human species barrier for prion transmission. Notably, in humans with high consumption of red meat, Neu5Gc incorporates metabolically into human cells and induces antibody responses against Neu5Gc (Samraj et al., 2015). While incorporation of Neu5Gc increases the likelihood of systemic inflammation (Samraj et al., 2015), antibodies against Neu5Gc might be beneficial for neutralizing prion infection of zoonic origin in humans. A second important aspect to consider is the functional consequences of enhanced sialylation of foreign PrP^Sc^ in SLOs (Srivastava et al., 2015). Enhanced sialylation in human SLOs could “humanize” prions of animal origin transmitted to humans by decorating them with Neu5Ac and helping to deceive the human immune system (Srivastava et al., 2015). A third interesting aspect is related to human-specific differences in the binding sites of human Siglecs for selective recognition of Neu5Ac over of Neu5Gc (Varki, 2010). Siglecs are a family of sialic acid-binding proteins with a number of important functions (reviewed in Rabinovich and Croci, 2012). While interactions between prions and Siglecs have not yet been documented (Bradford et al., 2014), such a possibility should not be excluded considering the large number of Siglecs expressed in humans and mice. Human-specific differences in Siglecs for selective recognition of Neu5Ac over of Neu5Gc are also important for the critical assessment of results obtained in humanized mice (mice expressing the human PrP gene). Humanized mice have often been used to assess susceptibility of humans to prion strains of animal origin or to model human-to-human transmission (Collinge et al., 1995; Asante et al., 2002; Wadsworth et al., 2004; Bishop et al., 2006; Cassard et al., 2014). Because humanized mice express mouse but not human Siglecs, interaction between Siglecs and PrP^Sc^ are expected to lead to different outcomes in humanized mice and in humans.

## Sialylation of PrP^Sc^ and lymphotropism

Prion strains show variable degrees of lymphotropism (Aguzzi et al., 2013). The molecular mechanism behind strain-specific lymphotropism is not known. It is also not known whether limited lymphotropism is due to deficient trafficking of certain strains to SLOs, impaired replication in SLOs, fast clearance in SLOs, or a combination of these factors. Recent studies revealed that sialylation of N-linked glycans at α2-6 linkages is responsible for directed trafficking and selective adhesion of hepatocarcinoma cells to SLOs (Zhang et al., 2013; Wang et al., 2015). Another work that employed synthetic glycoclusters demonstrated that in circulation the glycoclusters with α2-6 linked sialic residues were more stable and showed slower clearance rates in comparison to the glycoclusters with α2-3 linkages (Tanaka et al., 2010). It would be interesting to test whether sialylation and, in particular, α2-6 linkages also account for the lymphotropism of PrP^Sc^.

Notably, the two types of human CJDs, sporadic and variant, show significant differences with respect to their lymphotropism, with variant CJD known to be much more lymphotropic than sCJD (Hill et al., 1999; Wadsworth et al., 2001; Halliez et al., 2014). PrP^Sc^ in variant CJD is predominantly diglycosylated and, as such, more sialylated than PrP^Sc^ in sCJD, which is predominantly monoglycosylated (Zanusso et al., 2004; Pan et al., 2005). The relative ranking of the two types of CJD with respect to sialylation is consistent with the hypothesis that sialylation is important for effective trafficking of PrP^Sc^ to SLOs.

## Sialylation of PrP^Sc^ and toxicity

Interaction of PrP^Sc^ particles with PrP^C^ molecules anchored via GPI on the cell surface is believed to be important for triggering toxic signals (Solforosi et al., 2004; Sonati et al., 2012). Previous studies proposed that PrP^Sc^-induced toxicity is mediated via PrP^C^ molecules with sialylated GPI anchors (Bate and Williams, 2012a,b). Clustering of PrP^C^ with sialo-GPIs was shown to trigger synapse damage via activating cytoplasmic phospholipase A2 in neurons cultured *in vitro* (Bate and Williams, 2012a,b).

It is not known whether the toxic potential of PrP^Sc^ depends on sialylation status of its N-linked glycans. Considering that two positively charged regions in PrP are involved in mediating toxic signals (Solomon et al., 2011; Westergard et al., 2011), it is plausible that the binding between PrP^Sc^ and PrP^C^ involves electrostatic interactions between negatively charged sialic acid residues of PrP^Sc^ N-linked glycans and two solvent-exposed, positively charged regions of PrP^C^ (Turnbaugh et al., 2012). In agreement with this hypothesis is the observation that colominic acid, which is a polymer of sialic acid, blocks neurotoxicity of PrP^Sc^ toward cortical neurons cultured *in vitro* (Ushijima et al., 1999). Other support of this hypothesis comes from studies where abnormal, self-replicating PrP states referred to as atypical PrPres were found to exhibit very low sialylation levels of N-glycans and lack of toxicity in animal studies (Kovacs et al., 2013; Makarava et al., 2015). Atypical PrPres was fully transmissible in animal bioassays and accumulated in the form of small synaptic deposits and large plaques. However, atypical PrPres alone, in the absence of PrP^Sc^, did not cause neuronal death, pathological lesions or any clinical signs of prion diseases (Makarava et al., 2011, 2012a, 2015, 2016; Kovacs et al., 2013).

## Sialylation and strain interference

Prion strain interference occurs when a host is infected with two or more prion strains (Dickinson et al., 1972; Kimberlin and Walker, 1985; Bartz et al., 2007; Schutt and Bartz, 2008; Shikiya et al., 2010). Strain interference refers to an extension of the incubation time to disease produced by a strain mixture relative to the incubation period produced alone by the strain with the shortest incubation time. Among factors that were previously discussed as main contributors to strain interference were competition between strains for substrate, cellular co-factors, or cellular replication sites (Bartz et al., 2007; Shikiya et al., 2010).

Strain-specific selection of PrP^C^ sialoglycoforms adds an important dimension to the strain interference phenomenon (Katorcha et al., 2015b). Strains with substantial structural constraints rely on a narrow range of PrP^C^ sialoglycoforms as substrates and are unlikely to be strong competitors (Figure 4). In contrast, strains capable of recruiting a broad range of PrP^C^ sialoglycoforms have a greater chance of succeeding in competition for substrate. Evolution of prion diseases of synthetic origin and competition between two self-propagating states, atypical PrPres and PrP^Sc^ provides remarkable illustrations of how differences in selectivity toward PrP^C^ sialoglycoforms determined the outcome of competition (Makarava et al., 2011, 2012a, 2013, 2016). Only a small fraction of PrP^C^ sialoglycoforms that were acceptable as a substrate for PrP^Sc^ was found to be also a suitable substrate to atypical PrPres (Makarava et al., 2015). As a result, atypical PrPres replicated slower than PrP^Sc^, and PrP^Sc^ outcompeted atypical PrPres (Makarava et al., 2011, 2012a, 2015, 2016). In conclusion, strain-specific selection of PrP^C^ sialoglycoforms is an important factor that contributes to strain competition and interference.

## Role of sialylation in normal function of PrP^C^

The role of sialylation in the normal function of PrP^C^ has yet to be explored. PrP^C^ contains Lewis X [trisaccharide Galβ1-4(Fucα1-3)GlcNAc, abbreviated as Le^x^] and sialyl-Lewis X [tetrasaccharide NeuNAcα2-3Galβ1-4(Fucα1-3)GlcNAc, abbreviated as sLe^x^] epitopes (Stimson et al., 1999) that are known to serve as ligands for selectins. PrP^C^ containing Le^x^ epitopes were found to bind E-, L-, and P-selectins with nanomolar affinities and in a Ca^2+^ dependent manner (Li et al., 2007). A variety of biological activities involving PrP^C^, including neurotrophic activities (Chen et al., 2003; Santuccione et al., 2005; Lima et al., 2007), involvement in cell adhesion (Schmitt-Ulms et al., 2001; Santuccione et al., 2005; Viegas et al., 2006; Málaga-Trillo et al., 2009), and cell proliferation and differentiation (Mouillet-Richard et al., 1999; Steele et al., 2006; Zhang et al., 2006; Lima et al., 2007; Lee and Baskakov, 2010, 2013; Panigaj et al., 2011; Santos et al., 2011), has been observed over the years. In particular, a growing number of studies have highlighted the role of PrP^C^ in controlling self-renewal, proliferation and differentiation of stem cells, including human stem cells (Mouillet-Richard et al., 1999; Steele et al., 2006; Zhang et al., 2006; Lima et al., 2007; Lee and Baskakov, 2010, 2013, 2014; Panigaj et al., 2011; Santos et al., 2011). Considering that the proportion of di- vs. mono-, and unglycosylated PrP^C^ glycoforms increases in the course of neuronal differentiation and with the density of cells cultured *in vitro* (Monnet et al., 2003; Novitskaya et al., 2007), it is plausible that PrP^C^ glycosylation and sialylation is important for its function. Notably, recent studies revealed that deficiency in PrP^C^ in a cell resulted in a loss of polysialylation of Neural Cell Adhesion Molecule 1 (NCAM1) (Mehrabian et al., 2015). The defect in polysialylation was found to be due to impairment in expression of sialyltransferase ST8Sia2, which is responsible for polysialylating glycoproteins.

## Conclusions

Recent studies suggest that sialylation of PrP^Sc^ controls its fate in an organism and the outcomes of prion disease. PrP^Sc^ with reduced sialylation status did not cause prion disease presumably due to an increase in the amounts of terminal galactose that is believed to serve as “eat me” signal. The following mechanisms that define the sialylation of PrP^Sc^ have been identified: (i) sialylation of PrP^C^ by STs, (ii) strain-specific selective recruitment of PrP^C^ sialoglycoforms, and (ii) post-conversion enhanced sialylation of PrP^Sc^ in SLOs (Figure 3). In addition, sialylation of N-linked glycans was shown to contribute to the replication barrier that defines the rates of prion replication within the same host and prion transmission between different species. PrP^C^ with glycans of small sizes and fewer sialic acid residues per PrP^C^ molecule are expected to have a lower energy barrier for conversion relative to the heavily sialylated PrP^C^ with bulky glycans. For explaining strain-specific differences in glycoform ratios, selective exclusion of heavily sialylated PrP^C^ molecules from conversion due to strain-specific structural constraints was proposed. Nevertheless, because sialylation protects PrP^Sc^ against clearance and might be also important for prion transmission, lymphotropism and toxicity, to be highly infectious prion strains have to accommodate certain levels of sialylation. The precise role of sialylation in animal-to-human prion transmission, prion lymphotropism, toxicity, strain interference, and normal function of PrP^C^, have yet to be addressed and require future studies.

## Author contributions

IB wrote the manuscript; EK contributed to the section “N-linked glycans and PrP^Sc^ structure,” generated the models presented on Figure 3 and provided critical feedback.

## Funding

Financial support for this study was provided by National Institute of Health Grants R01 NS045585 and R01 NS074998.

### Conflict of interest statement

The authors declare that the research was conducted in the absence of any commercial or financial relationships that could be construed as a potential conflict of interest.
